# Kaposi Sarcoma Herpes Virus Latency Associated Nuclear Antigen Protein Release the G2/M Cell Cycle Blocks by Modulating ATM/ATR Mediated Checkpoint Pathway

**DOI:** 10.1371/journal.pone.0100228

**Published:** 2014-06-27

**Authors:** Amit Kumar, Sushil Kumar Sahu, Suchitra Mohanty, Sudipta Chakrabarti, Santanu Maji, R. Rajendra Reddy, Asutosh K. Jha, Chandan Goswami, Chanakya N. Kundu, Shanmugam Rajasubramaniam, Subhash C. Verma, Tathagata Choudhuri

**Affiliations:** 1 Division of Infectious Disease Biology, Institute of Life Sciences, Bhubaneswar, India; 2 Department of Biotechnology, Siksha Vhabana, Visva Bharati, Santiniketan, Bolpur, India; 3 School of Biotechnology, KIIT University, Bhubaneswar, India; 4 Department of Microbiology and Immunology, University of Nevada, Reno, School of Medicine, Reno, Nevada, United States of America; 5 School of Biological Science, National Institute of Science Education and Research, Bhubaneswar, India; 6 Department of Biotechnology, Regional Medical Research Centre for Tribals, Jabalpur, India; National Center for Cell Science, India

## Abstract

The Kaposi's sarcoma-associated herpesvirus infects the human population and maintains latency stage of viral life cycle in a variety of cell types including cells of epithelial, mesenchymal and endothelial origin. The establishment of latent infection by KSHV requires the expression of an unique repertoire of genes among which latency associated nuclear antigen (LANA) plays a critical role in the replication of the viral genome. LANA regulates the transcription of a number of viral and cellular genes essential for the survival of the virus in the host cell. The present study demonstrates the disruption of the host G2/M cell cycle checkpoint regulation as an associated function of LANA. DNA profile of LANA expressing human B-cells demonstrated the ability of this nuclear antigen in relieving the drug (Nocodazole) induced G2/M checkpoint arrest. Caffeine suppressed nocodazole induced G2/M arrest indicating involvement of the ATM/ATR. Notably, we have also shown the direct interaction of LANA with Chk2, the ATM/ATR signalling effector and is responsible for the release of the G2/M cell cycle block.

## Introduction

The Kaposi's sarcoma-associated herpesvirus (KSHV), or human herpesvirus-8 is a member of gammaherpes virus family and is etiologically associated with Kaposi's sarcoma (KS) [1], primary effusion lymphoma (PEL) [2], and a subset of multicentric Castleman's disease (MCD) [3]. This virus can infect a variety of human cell types such as cells of epithelial, mesenchymal and endothelial origin [4]. Generally they maintain latency in host cells characterized by the persistence of the viral genome as circular episome with limited viral gene expressions such as viral FLICE inhibitory protein (v-FLIP), viral cyclin (v-cyclin) and latency associated nuclear antigen (LANA) [5], [6]. These viral antigens are involved in modulating the host cell functions for its survival. In PEL, the host cells are dependent on KSHV for their long term survival, as loss of the KSHV genome results in their death suggesting the involvement of virus in manipulating host gene functions [7]. LANA is encoded by the open reading frame (ORF) 73 of KSHV and is expressed in KSHV infected cells and associated diseases [8], [9], [10]. This latent protein engages itself in contributing to viral persistence and tumorigenesis through chromosome tethering, DNA replication, gene regulation, anti-apoptosis and cell cycle regulation [11], [12], [13], [14], [15], [16]. LANA interacts with several transcription factors like E2F, Sp1, RBP-Jk, ATF4, Id-1, and Ets and causes their transcriptional activation [17], [18], [19], [20], [21], [22], while it represses mSin3A, CBP, RING3, GSK-3b and p53 [12], [23], [24], [25].

In general, the cell cycle is driven by the sequential activation of a series of cyclins and their catalytic subunits, the cyclin dependent kinases (CDKs). The timing of the activation of the different CDK isoforms determines the order of occurrence of the major cell cycle phases: G1 phase, S phase and G2/M phase [26]. The regulatory pathways that control activation of CDKs are known as checkpoints [27]. Disruption of these checkpoint controls are commonly encountered in cancerous cells and cells infected with DNA transforming viruses, which include adenovirus, simian virus 40, papillomavirus and Epstein Barr virus [28], [29], [30], [31], [32], [33], [34], [35]. Targeting cell cycle is a thrust area of research in drug development against cancer [36], [37]. Nocodazole is a common drug known to interfere with the polymerization of microtubule and cause G2/M arrest [38]. A large number of immortalized tumour cell lines are defective for this checkpoint arrest and are consequently sensitive to killing by nocodazole [39]. So, we tested the effect of this drug on KSHV positive cells and found that the virus is capable of releasing the nocodazole induced G2/M checkpoint arrest. Earlier the role of different KSHV encoded molecules on cell cycle regulation have also been reported such as v-cyclin induces entry of quiescent or G1-arrested cells to S-phase and deregulates mitotic progression [40], v-FLIP induces cellular transformation via NF-κB activation [41], and NF-κB promotes cell growth through cyclin D1 up regulation [42]. LANA is also known to inhibit host cell cycle arrest by interacting or modulating various host factors [43], [44], [45], [46]. It directly interacts with the short variant of BRD4 and releases the BRD4- and BRD2/RING3 induced G1 checkpoint arrest [43]. Further, it protects lymphoid cells from p16 INK4A induced cell cycle arrest and induces S-phase entry [44]. Deregulation of cell cycle check point may lead to tumorigenic events during which the ataxia telangiectasia mutated (ATM)/ATM Rad3- related (ATR) regulated checkpoint act as a guard against tumour progression. Check point kinases, Chk1 and Chk2 are downstream to ATM/ATR pathway and the roles of these two molecules in response to nocodazole treated cells are important, as inhibition of the Chk2 pathway results in a loss of the G2/M checkpoint [47]. Thus in order to ascertain the mechanism by which KSHV compromises cell cycle checkpoints and possible mechanistic involvement of LANA in releasing G2/M block were investigated. This study demonstrates a novel function of the LANA, in releasing the G2/M checkpoint arrest and its interaction with Chk2 to modulate the ATM/ATR signalling pathway.

## Materials and Methods

### Cell culture

The KSHV negative B-cell line, BJAB [17] and the KSHV positive B-cell line, BC3 [13] were provided by Prof. Erle S. Robertson, University of Pennsylvania, USA. Another KSHV positive B-cell line, JSC-1 [48], was provided by Prof. David J. Blackbourn, University of Surrey, UK. These cells were grown in RPMI-1640 medium (cat no. P04-16500) [Pan Biotech, GmbH, Germany]. The adherently growing human embryonic kidney cell line, HEK-293 [17] was grown in Dulbecco's modified eagle medium, DMEM (cat no. P04-20350) [Pan Biotech, GmbH, Germany]. The growth media were supplemented with 10% fetal bovine serum (Australian origin), 20 units/mL penicillin, 100 µg/mL streptomycin (Sigma, USA). Cultures were maintained in a humidified incubator at 37°C and 5% CO_2_.

### Antibodies

Polyclonal rabbit anti-phospho-Tyr15 Cdc2, mouse monoclonal Cdc2, mouse monoclonal cyclin B1, anti-HA rabbit polyclonal antibody, anti-Myc mouse monoclonal antibody, rabbit polyclonal anti-β–actin, HRP conjugated mouse and rabbit polyclonal antibodies were all purchased from Santa Cruz Biotechnology (USA), IMGENEX Corporation (USA) or Cell Signaling Technology, Inc. (USA).

### Plasmid constructs and siRNA

The pCDNA3.1-HA, pCDNA3.1-HAChk2, truncated Chk2 constructs cloned in pGEX2T, pA3M and pA3M-LANA expression vectors were provided by Prof. Erle S. Robertson, University of Pennsylvania, USA.Sequences of control siRNA, ATM siRNA, ATR siRNA and Chk2 siRNA were purchased from Santa Cruz Biotechnology (USA).

### Transfection

Transfection of expression vector was performed with LipofectAMINE Plus reagent according to the manufacturer's instructions (Invitrogen Inc) or by electroporation using a Bio-Rad Gene Pulser II electroporator [49]. Briefly, for electroporation 10 million cells were taken, washed in phosphate buffered saline (PBS) and then resuspended in 400 µl of either DMEM or RPMI 1640 according to the cell type. Resuspended cells were transferred to 4 mm electroporation cuvettes and electroporated. After electroporation the cells were plated in 10 mL supplemented media and grown at 37°C with 5% CO_2_ for 24 hours prior to harvesting. siRNA transfection was carried out following the manufacturer's instructions (Invitrogen Inc).

### Immunoprecipitation and Western blotting

Transfected cells were harvested, washed in PBS and lysed in 0.5 mL ice-cold radioimmunoprecipitation assay (RIPA) buffer [0.5% NP-40, 10 mM Tris pH 7.5, 2 mM EDTA, 150 mM NaCl and protease inhibitors]. Cellular debris was removed by centrifugation (21000 g, 10 min at 4°C) and the supernatant was transferred to a fresh tube. Approximately 5% of the cell lysate was saved as an input control. Lysates were then precleared with isotype control and then rotated with 30 µL of a 1∶1 mixture of protein A- and protein G-conjugated sepharose beads for an hour, at 4°C. Beads were spun out and the supernatant was transferred to a fresh microcentrifuge tube and the protein of interest was captured by rotating overnight with 1 µg of specific antibody at 4°C. Complexes were precipitated with 30 µL of 1∶1 mixture of protein A- and protein G-sepharose beads. The samples were then washed 3 times with ice-cold RIPA buffer, fractionated by SDS-PAGE and transferred onto a 0.45 µm nitrocellulose membrane for western blotting. The membranes were probed with the appropriate primary antibodies followed by incubation with appropriate HRP-tagged secondary antibodies. After further washing the membrane was developed using Luminol reagent Santa Cruz Biotechnology (USA).

### Glutathione S-transferase (GST) fusion protein preparation, *in vitro* binding assays and cellular lysate binding

The BL21 strain of *Escherichia coli* cells were transformed with pGEX2T-Chk2 plasmids (expressing 4 different truncations of Chk2) and selected on an ampicillin plate. Bacterial culture grown overnight from a single colony was inoculated into 500 mL of Luria-Bertani medium and grown to log phase with shaking at 37°C. The cells were then induced with 1 mM isopropyl-β-_D_-thiogalactopyranoside (IPTG) overnight with shaking at 30°C. The cells were then harvested, sonicated, and then the protein was solubilized. The cell lysate so obtained was then incubated with glutathione-sepharose beads overnight with rotation at 4°C. The beads were then collected by centrifugation and washed 3 times with NETN buffer (20 mM Tris HCl of pH 8.0,100 mM NaCl, 1 mM EDTA and 0.5% Nonidet P-40 and protease inhibitors). The protein-bound beads were stored at 4°C in NETN buffer. The full-length pA3M-LANA clone was translated *in vitro* with [^35^S] methionine-cysteine in the T7 TNT system (Promega, USA). The *in vitro*-translated proteins were also precleared with glutathione-sepharose beads in binding buffer (1×PBS, 0.1% NP-40, 0.5 mM dithiothreitol, 10% glycerol and protease inhibitors) for 30 minutes with rotation at 4°C, and the beads were removed by centrifugation. The precleared proteins were then incubated with different truncated constructs of GST-Chk2 for 24 hours with rotation at 4°C. Then, the beads were pelleted by centrifugation and washed 3 times with the binding buffer. The bead bound protein were then mixed with laemmli buffer and boiled for 5 minutes, followed by SDS-polyacrylamide gel electrophoresis (PAGE).

The KSHV positive cells nuclear extract used in the binding experiments was prepared as described previously [49]. The nuclear extract was precleared with glutathione-sepharose beads for 30 minutes with rotation at 4°C. Again, the lysates were precleared with GST-bound glutathione-sepharose beads for one hour with rotation at 4°C. Then, the lysates were incubated with GST-Chk2 bound beads, and rotated overnight at 4°C. SDS lysis buffer with heating was used to elute the bound protein from the beads and subjected to SDS-PAGE and detected for LANA by immunoblotting.

### Flow cytometric analysis of cell cycle phase distribution of nuclear DNA

For the determination of the cell cycle phase distribution of nuclear DNA, cells were harvested, fixed with 70% ethanol, permeabilized with 0.5% Triton X-100, and nuclear DNA was stained with propidium iodide (PI; 25 µg/mL) after RNase treatment. The cell cycle analysis was determined with a FACS Calibur (Becton Dickinson) using Cell Quest software (BD Biosciences, USA). Histograms displaying DNA content (as indicated by PI fluorescence; x axis) versus counts (y axis) were created. To quantitate the data at different phases of the cell cycle, a total of 10,000 events were acquired.

### Immunofluorescence

Cells were fixed in 4% paraformaldehyde and then blocked in the appropriate serum prior to incubation with mouse monoclonal anti-Myc for LANA and rabbit polyclonal anti-HA for Chk2 for an hour. Cells were washed and then further incubated with the appropriate secondary antibody conjugated to Alexa Fluor 488 (green) and Alexa Fluor 594 (red) at 1∶500 dilutions in PBS for an hour. Slides were then washed, and visualized under a laser scanning confocal-microscope (Leica TCS SP5, Germany).

## Results

### The KSHV latent antigen LANA releases the nocodazole induced G2/M cell cycle block in human B-cells

**Figure 1:**
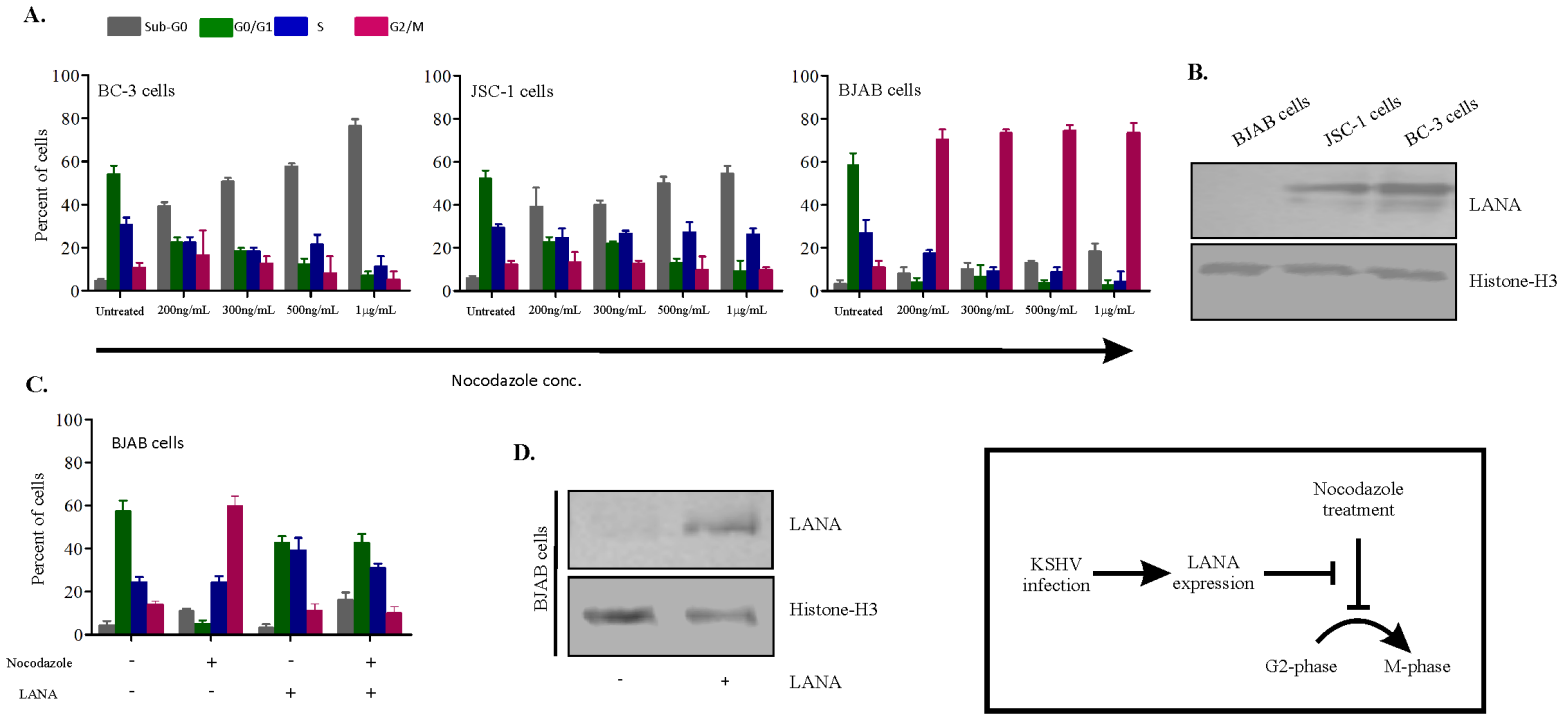
Cell cycle profiles of KSHV positive and negative cells treated with nocodazole showed that KSHV is capable of releasing nocodazole induced G2/M block though LANA. (A) Two KSHV positive cells (BC3 and JSC-1), and a KSHV negative cells (BJAB) were treated with or without different concentration of nocodazole (200 ng/mL, 300 ng/mL, 500 ng/mL or 1 µg/mL) for 24 hours. The cells were then harvested, stained with PI and their cell cycle profiles were determined by flow cytometry. Nocodazole treated BJAB cells showed an increase in the proportion of cells at G2/M, indicating a block, whereas BC3 and JSC-1 showed no block in G2/M phase. Results are indicated as percentages of cells in all cell cycle phases. Mean and standard deviations were derived from 3 independent experiments. (B) Western blot shows the expression of LANA in KSHV positive cells (BC-3 and JSC-1) but not in KSHV negative cells (BJAB). Histone-H3 was taken as loading control. (C) BJAB cells transfected with the pA3M vector or pA3M-LANA and were treated with nocodazole (200 ng/mL) for 24 hours. The cells were then harvested and DNA cell cycle distribution patterns were determined as above. Results in the untreated and treated cells are shown as the percentage of cells in all cell cycle phases. Mean and standard deviations were derived from 3 independent experiments. (D) Western blot shows the expression of LANA in the BJAB cells transfected with pA3M-LANA. Histone-H3 was taken as loading control. The result shown is the representative of 3 independent experiments. +, present; −, absent.

The KSHV positive cells (BC3 and JSC-1), were treated with nocodazole in a dose-dependent manner for 24 hours, and their cell cycle phases were determined by flow cytometry (Fig. 1A, left and middle panels). Untreated control cells showed a normal cell cycle distribution pattern. Nocodazole treatment causes G2/M-arrest in BJAB cells (Fig. 1A, right panel). On the other hand no G2/M check point arrest was noted in nocodazole treated BC3 or JSC-1 cells at all concentration tested (200 ng/mL, 300 ng/mL, 500 ng/mL or 1 µg/mL)[Fig. 1A, left and middle panels]. These results suggest that the KSHV positive cell are resistant to and/or reverse nocodazole induced G2/M block and allow cell cycle progression. Additionaly, increase in concentration of the nocodazole lead to increase in sensitivity of the cells to cytotoxic effect of the drug (data not shown). To confirm the presence of KSHV in BC3 and JSC-1 cells, KSHV latent antigen LANA was tested. Figure 1B shows the presence of LANA in these cells. As KSHV reversed the nocodazole induced G2/M checkpoint, it was interesting to investigate which of the KSHV latent nuclear proteins were responsible for this effect. Since BJAB cells were susceptible to G2/M arrest, it was a suitable cell line for testing the effects of KSHV latent proteins. Previously involvement of LANA in release of G1 cell cycle arrest has been indicated [43], therefore the role of this viral protein on the G2/M check point was evaluated. BJAB cells transfected with the empty vector alone (pA3M vector) or LANA expressing vector (pA3M- LANA) were treated with nocodazole (200 ng/mL) for 24 hours (Fig. 1C). Nocodazole treatment induces the G2/M arrest in pA3M vector transfected BJAB cells but the expression of LANA alone without nocodazole did not significantly affect the normal cell cycle distribution pattern. In contrast, nocodazole failed to elicit G2/M arrest in BJAB (pA3M- LANA) (Fig. 1C and D). This response was nearly similar to that observed in KSHV positive cells. These results indicate that LANA is effective in releasing the nocodazole induced G2/M cell cycle block and allows cell cycle progression.

### LANA inhibits the suppression of Cdc2 (Tyr15) phosphorylation triggered by nocodazole

**Figure 2:**
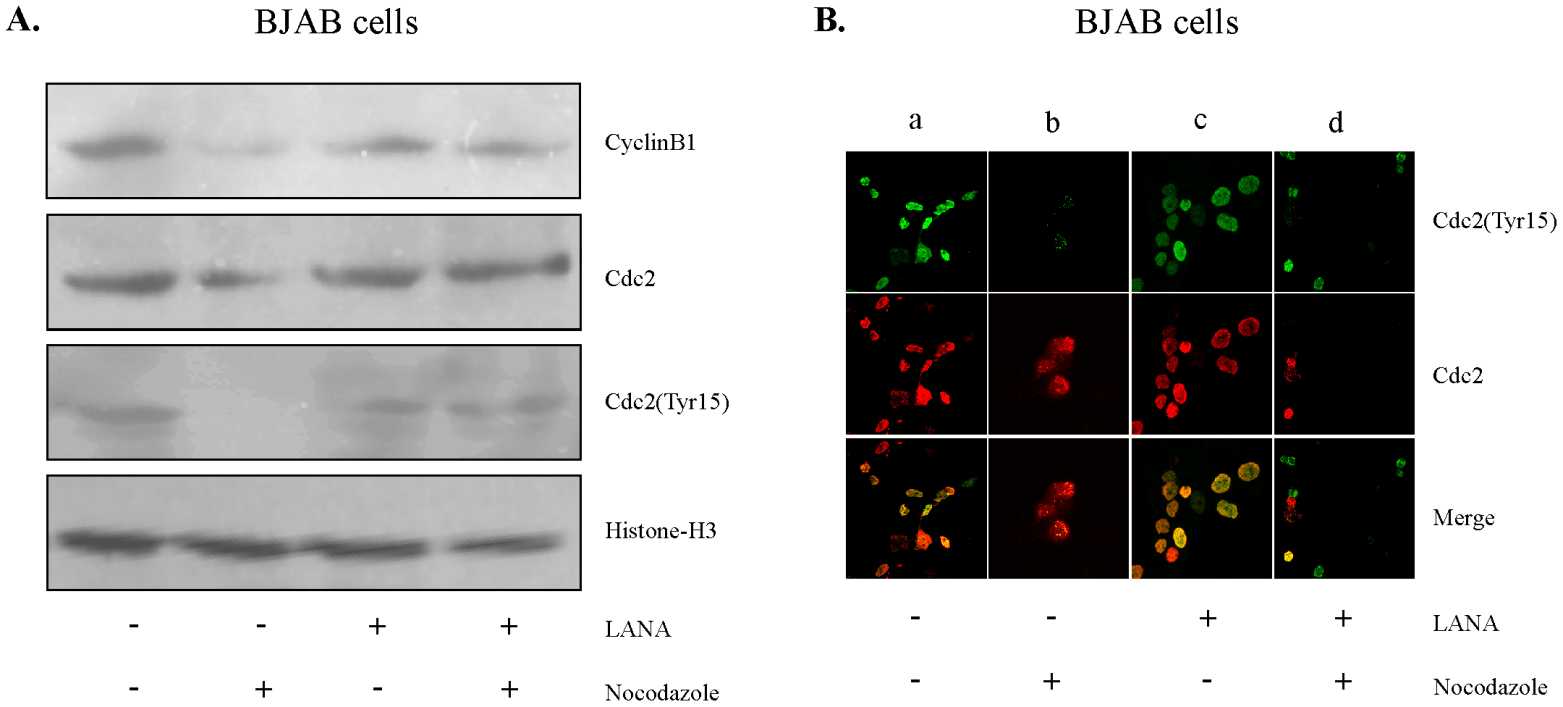
Nocodazole induced suppression of the Cdc2 (Tyr15) phosphorylation inhibited by the LANA expression. (A) BJAB cells transfected with the pA3M vector or pA3M-LANA were treated with nocodazole (200 ng/mL) for 24 hours. The cells were then harvested, total cell lysate were prepared and western blot was performed using antibodies against cyclin B1, Cdc2, Tyr15-phosphorylated Cdc2 [Cdc2 (Tyr15)], and Histone H3 as a protein loading control. (B) BJAB cells transfected with the pA3M vector or pA3M-LANA were grown in 6 well culture plates and treated with nocodazole and observed for colocolaization in a confocal microscope. The result shown is the representative of 3 independent experiments. +, present; −, absent.

Earlier studies have demonstrated involvement of cyclin B and Cdc2 in the blockage of the cell cycle machinery and consequent progression into mitosis [50], [51]. The G2/M checkpoint arrest is due to blocking of B-Cdc2 activation, detected as an accumulation of inactive form of Tyr15-phosphorylated cyclin B-Cdc2 complex [52]. Our studies have demonstrated nocodazole induced G2/M arrest in BJAB cells, therefore, the inhibition of the checkpoint response by LANA may be due to the disruption in activation of the cyclin B-Cdc2 complex and a consequent deficiency in accumulation of this inactive complex. To explore this effect, the levels of cyclin B1, Cdc2 and phosphorylated Cdc2 (Tyr-15) were analyzed by immunoblotting in nocodazole treated and untreated BJAB cells and LANA expressing BJAB cells (Fig. 2A). In LANA expressing cells, the levels of total cyclin B, Cdc2 and phosphorylated Cdc2 (Tyr-15) remained similar in the treated as well as untreated cells. But, in non-LANA expressing cells a dramatic change in the level of phosphorylated Cdc2 (Tyr15) was observed in treated cells (Fig. 2A). Interestingly, the initiation of mitosis depends upon the phosphorylation of Cdc2 at Tyr15 [52]. Furthermore, phosphorylation in Cdc2 (Tyr15) was drastically downregulated in BJAB cells in the presence of nocodazole, suggesting G2 arrest. In contrast, no detectable change in Cdc2 (Tyr15) phosphorylation was observed in BJAB cells expressing LANA after nocodazole treatment (Fig. 2A). To further corroborate these results, immunofluorescence assay was performed In Fig. 2B shows (a) The basal level expression of phosphorylated Cdc2 (Tyr15) (green fluorescence), Cdc2 (red fluorescence) and the merged image as visualized under confocal-microscope (b) Treatment of nocodazole in these cells causes reduction in phosphorylated Cdc2 (tyr 15) (green fluorescence) but no detectable change in Cdc2 (red fluorescence wa observed. (c) Expression of LANA alone in the absence of nocodazole id not affect the phospho Cdc2 (Tyr15) (green fluorescence), Cdc2 (red fluorescence). (d) Nocodazole induced reduction in phosphorylated Cdc2 (Tyr 15) (green fluorescence) was abrogated by LANA. The above results indicate involvement of LANA in relieving the G2 block by regulating Cdc2 (Tyr15) phosphorylation (Fig. 2A and B).

### LANA is capable of disrupting the nocodazole induced G2/M cell cycle arrest through the ATM/ATR signalling pathway

**Figure 3:**
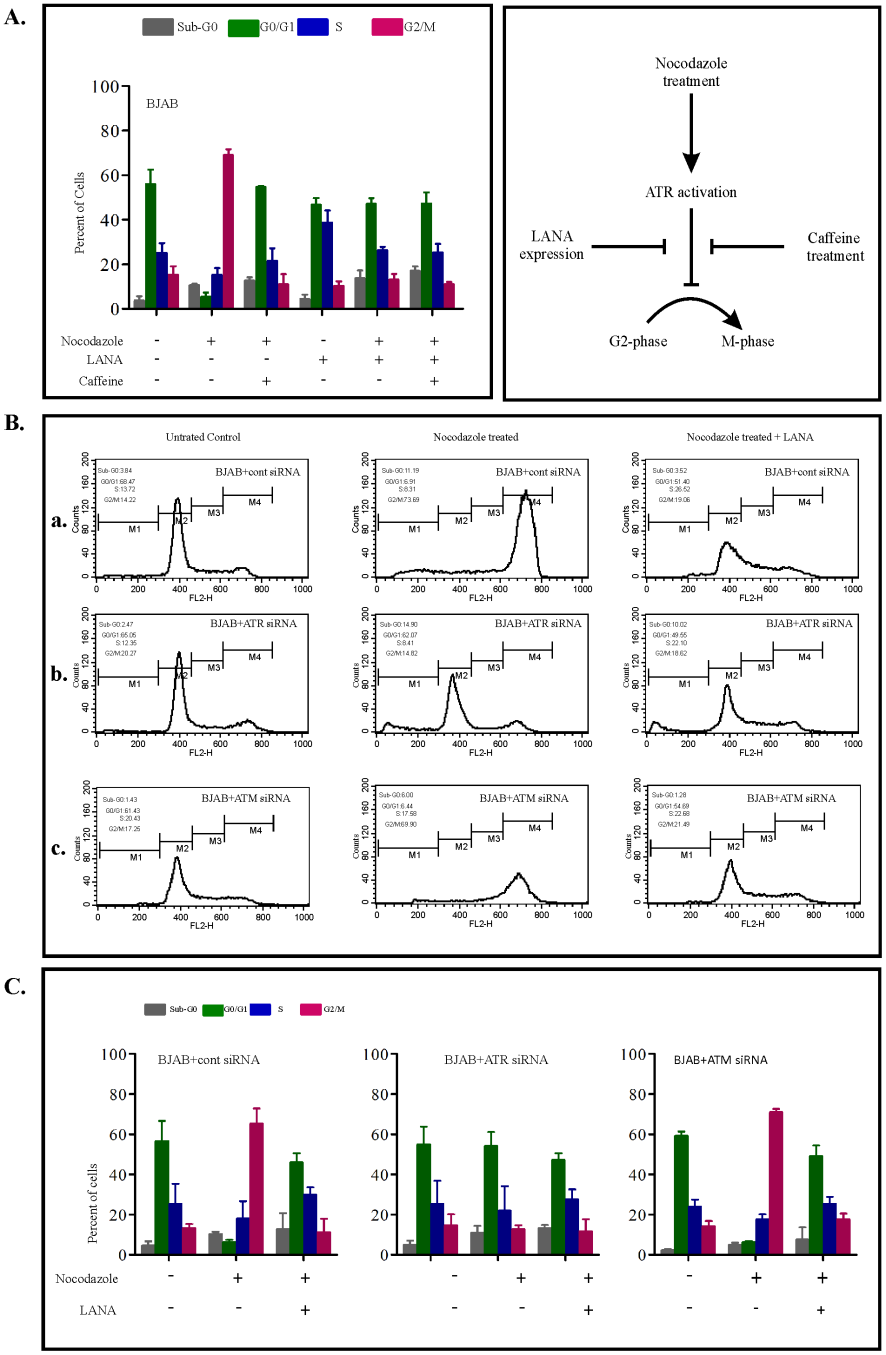
LANA disrupt the nocodazole induced G2/M cell cycle block through the ATM/ATR signaling pathway. (A) The release of nocodazole induced G2/M block by LANA is inhibited by caffeine. BJAB cells (transfected with the pA3M vector or pA3M-LANA) were treated with nocodazole (200 ng/mL) with and without caffeine (5 mM) for 24 hours. The cells were then harvested, stained with PI and their DNA cell cycle profiles were determined by FACS analysis. Results shown are percentages of cells in each phase of the cell cycle. The data represent the mean of 3 separate experiments. (B, C) The release of nocodazole induced G2/M block by LANA occurs through ATR. BJAB cells (control), ATM siRNA and ATR siRNA transfected BJAB cell were treated with nocodazole (200 ng/mL) in the presence and absence of LANA. The cells were then harvested stained with PI and their DNA cell cycle phases were determined by FACS. Results are shown as the percentages of cells in all cell cycle phases. Mean and standard deviations were derived from 3 independent experiments. +, present; −, absent.

Previous studies have shown that nocodazole and other cytotoxic drugs used for synchronizing cell cycle phases may affect the DNA damage response pathway, specifically the ATM/ATR pathway [35], [53]. This pathway is a network of interacting pathways that act at the G2 phase of the cell cycle to block G2/M progression by inhibiting the activation of cyclin B-Cdc2. The ability of LANA to inhibit the G2/M checkpoint blockage in response to nocodazole indicates that LANA may be targeting this pathway. This possibility was tested using the known sensitivity of both ATM and ATR to inhibition by caffeine [34]. In fact the ATM/ATR signalling was involved in nocodazole response, suggesting that this signalling pathway may be a target for regulation by LANA. Caffeine efficiently blocked the nocodazole induced G2 phase arrest in BJAB cells (Fig. 3A). Notably, the effect of caffeine on nocodazole induced cell cycle arrest was nearly similar to that of LANA (Fig. 3A). To address this effect, we used ATM siRNA, ATR siRNA transfected and untransfected BJAB cells and carried out cell cycle analysis. In Fig. 3B: (a) Nocodazole induced G2/M block in control siRNA transfected BJAB cell was released by LANA expression. (b) ATR siRNA transfected BJAB cells failed to release the nocodazole induced cell cycle arrest indicating their involvement in this process. LANA expression in these cells had no major effect on cell cycle pattern. (c) Response of ATM siRNA transfected cells was similar to control siRNA transfected BJAB cells and had no discernible effect on the cell cycle. The results provided in Fig. 3B were repeated in 3 independent experiments and is shown (Fig. 3C).

### siRNA mediated downregulation of Chk2 expression inhibits LANA mediated release of nocodazole induced G2/M cell cycle arrest

**Figure 4:**
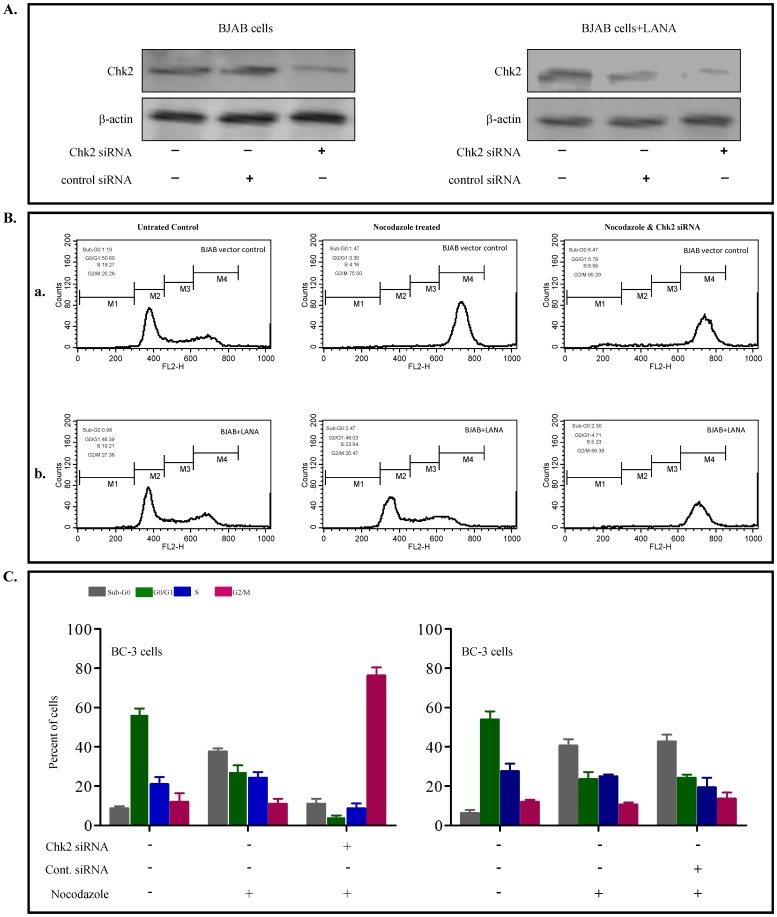
Downregulation of Chk2 protein levels inhibits the ability of LANA to release the nocodazole G2/M block. (A) Western blot for Chk2 showed the specific effect of Chk2 siRNA on Chk2 protein levels. BJAB cells carrying pA3M vector (control) and pA3M-LANA were transfected with control and Chk2 siRNA for another 24 hours. The cells were harvested, whole cell lysates and subjected to western blot analysis. β-actin was used as loading control. The result shown is the representative figure of 3 independent experiments. (B) BJAB cells with pA3M vector and pA3M-LANA were treated with nocodazole and with and without Chk2 siRNA for 24 hours. The cells were then harvested, stained with PI and their cell cycle phases were determined by flow cytometry. Results in the untreated and treated cells are shown as the percentages of cells in various cell cycle phases. (C) BC3 cells transfected with control or Chk2 siRNA were treated with nocodazole for 24 hours and subjected cell cycle analysis. Results in the untreated and treated cells were shown, together with the percentages of cells in all cell cycle phases. Mean and standard deviations were derived from 3 independent experiments. +, present; −, absent.

Chk2 is a downstream target of ATM/ATR signalling pathway and may be activated through DNA damage response to cause cell cycle arrest. Hence, to explore the role of Chk2 in the LANA mediated release of the G2/M phase block; we used siRNAs to downregulate Chk2 expression. Chk2 siRNA transfected cells showed lower Chk2 levels in both BJAB and LANA expressing BJAB cells as compared to siRNA controls cells (Chk2 expression remained unaffected in cells transfected with scrambled control siRNA (Fig. 4A). Effect of Chk2 siRNA on cell cycle in BJAB cells and BJAB-LANA cells were assessed 24 hours post nocodazole treatment. In Fig. 4B: (a) In control BJAB cells nocodazole induces G2/M block and suppression of Chk2 in these cells and showed no discernible effect to modulate the cell cycle pattern. (b) In BJAB-LANA cells suppression of Chk2 abrogated LANA mediated bypass of nocodazole induced G2/M block These results suggest that LANA may utilize Chk2 to release the G2/M cell cycle arrest.

We also tested the effect of Chk2 siRNA on BC3 cells on cell cycle progression in presence of nocodazole. BC3 cells with Chk2 siRNA showed an arrest in G2/M-phase (Fig. 4C, left panel) while cells with control siRNA showed no detectable change in cell cycle (Fig. 4C, right panel). Thus clearly indicating that downregulation of Chk2 levels facilitates nocodazole induced the G2/M block in KSHV positive BC3 cells, and the KSHV latent protein LANA may act through Chk2 to release the G2/M blocking.

### LANA interacts with Chk2 and co-localizes inside the nucleus of human B-cells

**Figure 5:**
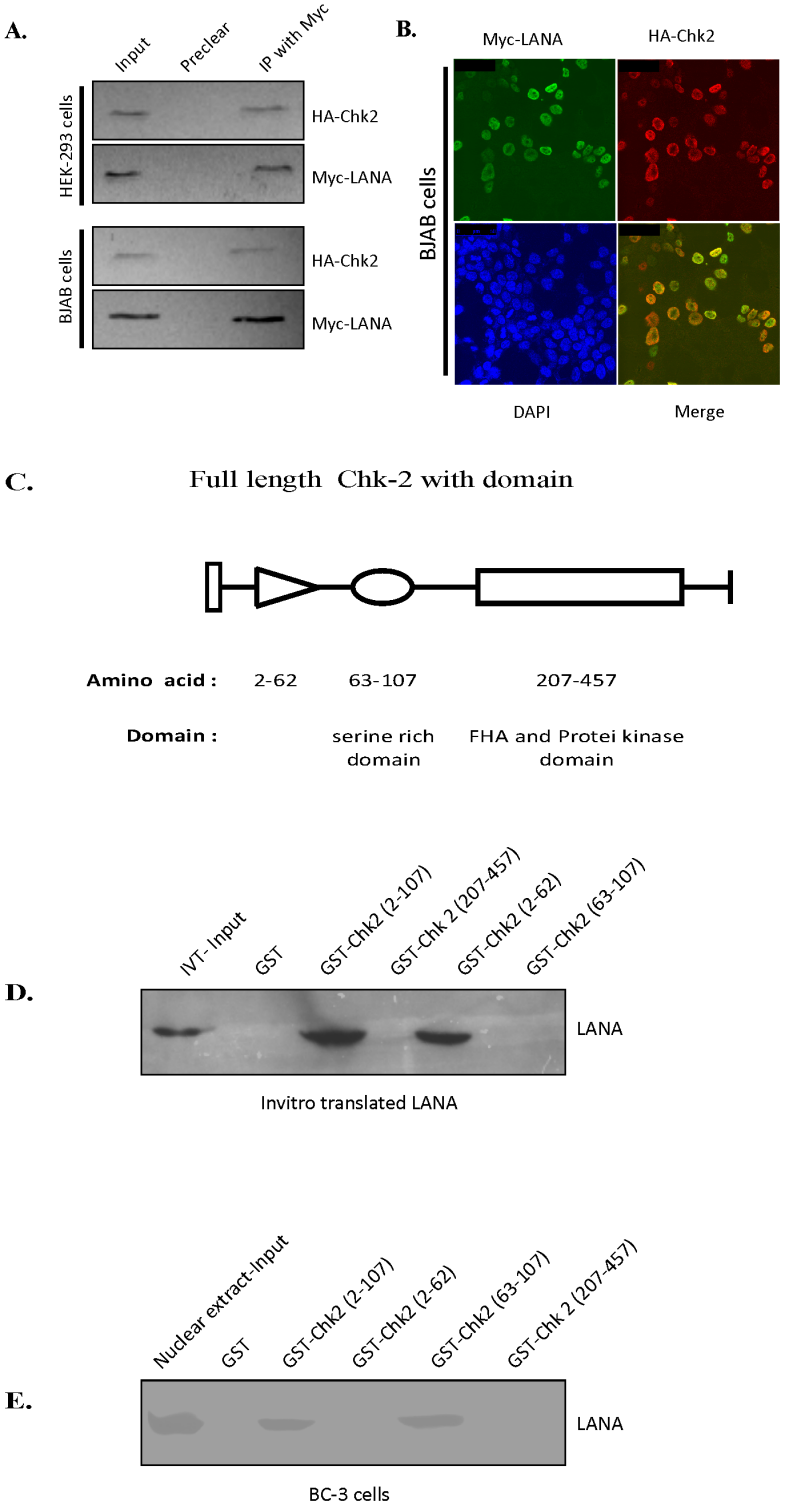
LANA interacts with serine rich amino-terminal domain of Chk2 inside the nucleus. (A) BJAB and HEK-293 cells were co-transfected with constructs pCDNA3.1-HAChk2 and pA3M-LANA. Co-immunoprecipitation from the cell lysate was performed by using anti-Myc antibodies. The co-immunoprecipitates were separated by electrophoresis, transferred to a nitrocellulose membrane, and then probed with HA antibodies for Chk2. Chk2 immunoprecipitated with LANA in both cell types. (B) BJAB cells were co-transfected with the expression constructs pCDNA3.1-HAChk2 and pA3M-LANA. Following transfection, the cells were grown overnight and fixed. LANA and Chk2 were detected by using mouse monoclonal antibody against Myc-LANA and rabbit polyclonal antibody against HA- Chk2, followed by appropriate secondary antibodies conjugated to Alexa Fluor 488 (green) and Alexa Fluor 594 (red), respectively. The merged panel shows that Chk2 and LANA co-localize in the nucleus. The DAPI panel shows that both proteins are nuclear. (C) Schematic representation of full-length domains along with the different truncation constructs of Chk2. FHA: fork head association domain. (D, E) *In vitro* translated LANA or KSHV-positive BC3 cells nuclear extract were incubated with the various GST-Chk2 truncated constructs as shown in figure. The pull-down assay showed a preferential binding for the region located between amino acids 63 and 107, which includes the serine rich domain. NE, nuclear extract.

To determine if there is any association between LANA and Chk2, co-immunoprecipitation was performed in BJAB and HEK-293 cells expressing LANA and Chk2. The Chk2 tagged with the HA epitope was found to be co-immunoprecipitated with Myc-tagged LANA from BJAB and HEK-293 cells as evidenced with anti-Myc antibodies in immunoprecipitation (Fig. 5A). This finding was corroborated and confirmed by immunofluorescence analysis in BJAB cells, which clearly showed the co-localization of these two molecules inside the nucleus (Fig. 5B).

### LANA binds to the serine rich amino-terminal domain of Chk2

Three domains of Chk2 protein (serine rich, FHA and protein kinase) are shown in schematic diagram (Fig. 5C). Efforts were made to map the binding region of LANA and for this ^35^S labeled LANA was incubated with a number of truncated mutants of GST-Chk2 polypeptide (Fig. 5D). The binding assay revealed that the Chk2 - LANA interaction region lies between amino acids 63 to 107, a serine rich domain of Chk2 (Fig. 5D). This region also overlaps with the fork head association (FHA) domain. Additionally, the data indicated that the carboxy-terminal region comprising amino acids 207 to 457 of Chk2 is not involved in binding, as there was no detectable interaction between this region and LANA (Fig. 5D). The same were confirmed with nuclear extracts from KSHV positive cells (BC3) expressing LANA supporting the *in vitro* data showing an association of LANA with the serine rich region of Chk2 (Fig. 5E).

## Discussion

The KSHV latency maintenance requires the coordinated and timely expression of a number of viral latent genes that function to rescue the rejection by the host immune response. The function of these individual viral genes, including the LANA, required during these events has been the focus of different research groups. Yet, the functional role of LANA in KSHV infected human cells remains to be completely elucidated. Previous studies have shown that LANA functions as a transcriptional regulator and modulates the function of different cellular genes [12], [17], [18], [19], [20], [21], [22], [23], [24], [25].

In the present studies, nocodazole failed to induce G2/M arrest in KSHV positive cells (Fig. 1A and B), a response similar to that previously seen in the tumour and transformed cell lines having a dysfunctional checkpoint [54]. However, nocodazole induces G2/M arrest in cells having a functional checkpoint response, but not in cells with a defective checkpoint. This finding suggests that KSHV positive cells may have a dysfunctional G2/M checkpoint. The present work revealed that expression of LANA protein may be critical for the release of nocodazole induced G2/M arrest (Fig. 1BC and D). This idea was supported by analysis of Cdc2 protein levels, which showed the recovery of active Cyclin B1-Cdc2 in the presence of LANA (Fig. 2). We have clearly shown that the G2/M checkpoint response to nocodazole is sensitive to inhibition by caffeine (Fig. 3A), implicating the role of ATM/ATR, and more specifically, ATR (Fig. 3B and C). One of the checkpoint kinases downstream of ATM/ATR is Chk2, and western blotting indicated that its expression may be downregulated by LANA. It is known that Chk2 is activated in response to G2/M initiating agents, such as the plant isoflavone genistein [55], and G2/M checkpoint is defective in Chk2 in embryonic stem cells [56], thus supporting a role for Chk2 in the G2/M checkpoint response. Therefore, LANA may be bypassing the nocodazole induced G2/M block by an alternate/indirect mechanism not linked to nocodazole mediated microtubule disruption.

**Figure 6:**
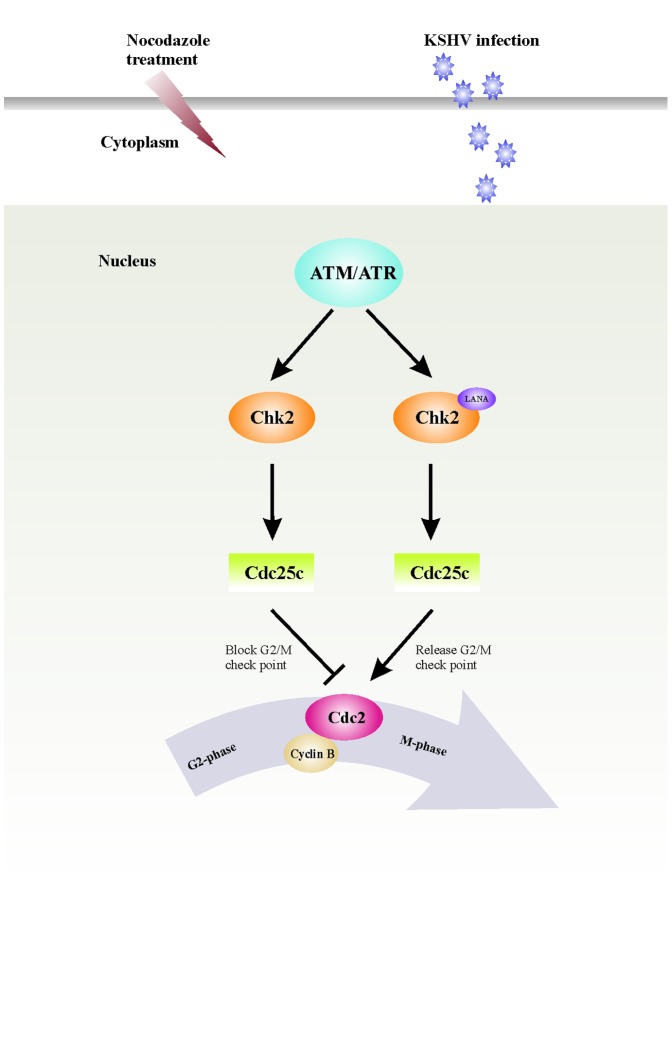
A hypothetical model shows the putative mechanisms for the bypassing of the nocodazole induced G2/M block by LANA. Nocodazole treatment reduces the level of phosphorylated Cdc2. The viral nuclear antigen LANA binds directly to Chk2, which may result in the phosphorylation of Cdc25c and sequester it in the cytoplasm. Thus, it may be unable to regulate the phosphorylation of nuclear Cdc2 resulting the activation of cyclin B-Cdc2 and progression through the G2/M phase, releasing the nocodozole induced block.

The physical interaction between LANA and Chk2 in LANA expressing BJAB cells suggests that LANA can disrupt the G2/M checkpoint response by directly blocking Chk2 function (Fig. 5A). This idea is supported by the findings that siRNA mediated downregulation of Chk2 diminished the ability of LANA in mediating the release of nocodazole induced G2/M arrest (Fig. 4). LANA and Chk2 co-localize inside the nucleus of BJAB cells (Fig. 5B) and we have demonstrated that LANA binds directly to the serine rich domain within the amino-terminal region of Chk2 (Fig. 5C, D and E). However, the functional relevance of this specific domain has not been understood, but it is likely that this domain may be regulated by LANA in KSHV-positive cells. Thus LANA binding to Chk2, an effector of the ATM/ATR signalling pathway may result in destabilization and increase in the turnover of Chk2, comparable to the effect of the E6 protein of human papillomavirus disrupting the auto-regulatory feedback loop p53 and MDM2 [57]. The Chk2 may cause phosphorylation of Cdc25c to sequester in the cytoplasm and render it ineffective to regulate the phosphorylation of nuclear Cdc2 resulting the activation of cyclin B-Cdc2 and progression through the G2/M phase, releasing the nocodozole induced block. Most interestingly, the present study has identified a novel role of LANA in its ability to disrupt the DNA damage and replication G2/M checkpoint and hence we put forth it as a basic mechanism for this activity (Fig. 6).

## Conclusions

The KSHV released the nocodazole induced G2/M cell cycle check point. The present studies clearly assign this critical role to LANA in executing this event. Importantly, LANA disrupts the cyclin B and Cdc2 mediated G2/M checkpoint response. Also, we have demonstrated that the treatment with caffeine abolished nocodazole induced the G2/M arrest, suggesting an involvement of the ATM/ATR signaling pathway in this regulation. Finally, we have shown that the physical interaction of LANA with ATR signaling effector, Chk2 inside the nucleus of B-cells is responsible for the release of nocodazole induced G2/M arrest.
